# Social and emotional relevance in face processing: happy faces of future interaction partners enhance the late positive potential

**DOI:** 10.3389/fnhum.2014.00493

**Published:** 2014-07-16

**Authors:** Florian Bublatzky, Antje B. M. Gerdes, Andrew J. White, Martin Riemer, Georg W. Alpers

**Affiliations:** School of Social Sciences, Clinical Psychology, Biological Psychology, and Psychotherapy, University of MannheimMannheim, Germany

**Keywords:** face processing, social interaction, emotion, anticipation, ERP

## Abstract

Human face perception is modulated by both emotional valence and social relevance, but their interaction has rarely been examined. Event-related brain potentials (ERP) to happy, neutral, and angry facial expressions with different degrees of social relevance were recorded. To implement a social anticipation task, relevance was manipulated by presenting faces of two specific actors as future interaction partners (socially relevant), whereas two other face actors remained non-relevant. In a further control task all stimuli were presented without specific relevance instructions (passive viewing). Face stimuli of four actors (2 women, from the KDEF) were randomly presented for 1s to 26 participants (16 female). Results showed an augmented N170, early posterior negativity (EPN), and late positive potential (LPP) for emotional in contrast to neutral facial expressions. Of particular interest, face processing varied as a function of experimental tasks. Whereas task effects were observed for P1 and EPN regardless of instructed relevance, LPP amplitudes were modulated by emotional facial expression and relevance manipulation. The LPP was specifically enhanced for happy facial expressions of the anticipated future interaction partners. This underscores that social relevance can impact face processing already at an early stage of visual processing. These findings are discussed within the framework of motivated attention and face processing theories.

## Introduction

Humans are intrinsically social. From an evolutionary perspective, social information is critical for survival as it contributes to successful commitment, procreation and preservation (Tooby and Cosmides, 1992; Brothers, 2002). Thus, conspecifics are primary elicitors of emotions designed to promote both affiliation and protection in the face of constantly changing environmental conditions (Keltner and Kring, 1998). Accordingly, viewing facial stimuli is highly informative and mediates perceptual, physiological, and behavioral responses (Hamm et al., 2003; Vuilleumier and Pourtois, 2007).

To investigate the link between social and emotional information processing, the present study focuses on the social relevance of facial pictures. Human faces contain salient social signals mediating information about one’s own and the others’ identity, emotional state, and intentions (Ekman and Friesen, 1975; Öhman, 1986). The neural signature of face processing has been outlined in recent research (Haxby et al., 2002; Adolphs and Spezio, 2006). Given the crucial importance of being able to efficiently read and understand facial expressions, it has been proposed that distinct brain structures are centrally involved in face processing (e.g., fusiform face area (FFA), superior temporal sulcus (STS); Kanwisher et al., 1997 but see Chao et al., 1999). In addition, research has identified several neural substrates involved in both emotional and social processes (e.g., amygdala, insular, medial prefrontal cortex (MPFC); Gusnard et al., 2001; Norris et al., 2004; Williams et al., 2004; Northoff et al., 2006; Schmitz and Johnson, 2007; Olsson and Ochsner, 2008; Sabatinelli et al., 2011). In order to adequately interact in social situations, observing emotional facial expressions facilitates perceptual, attentional, and behavioral responses (Alpers and Gerdes, 2007; Alpers et al., 2011). For instance, in visual search tasks, threatening (schematic) faces are detected more quickly than friendly or neutral target faces especially among highly anxious participants (Byrne and Eysenck, 1995; Öhman et al., 2001). In line with an evolutionary perspective, this processing advantage has been described specifically for angry and fearful faces mediating potential threat to the observer (Byrne and Eysenck, 1995; Whalen et al., 2001).

Electrophysiological measures are particularly well-suited to investigate the temporal dynamics of face processing. Event-related brain potential (ERP) studies have revealed processing differences for facial stimuli within the first 100 ms after stimulus onset. For instance, suggested to reflect attention gain control in extrastriate sources, enhanced P1 amplitudes were observed for fearful compared to neutral faces in visuo-spatial attention tasks (Pourtois et al., 2004). Further, temporo-occipital negativities have been shown to be sensitive to facial stimuli (N170; Bentin et al., 1996) and emotional facial expression (early posterior negativity, EPN; Schupp et al., 2004). The N170 is probably the most frequently investigated ERP component in face processing. It has been primarily related to the structural encoding of faces in temporo-occipital processing areas; for instance, as evidenced by studies manipulating structural features (e.g., face inversion; Itier and Taylor, 2002), presentation of specific face and body parts (e.g., only eye region; Itier et al., 2006), and spatial attention tasks (Holmes et al., 2003; Jacques and Rossion, 2007). Regarding the emotional state and intentions conveyed by facial expression, an early posterior negativity (occipito-temporal EPN; 150–300 ms) and late positive potentials (centro-parietal LPP; 300–700 ms) have been observed for angry as compared to neutral faces, but also for happy faces (Sato et al., 2001; Liddell et al., 2004; Schupp et al., 2004; Williams et al., 2006; Holmes et al., 2008; but see Wangelin et al., 2012). Further, these effects were more pronounced in socially anxious participants (Moser et al., 2008; Sewell et al., 2008; but see Mühlberger et al., 2009) and participants undergoing socially-mediated aversive anticipation (Wieser et al., 2010; Bublatzky and Schupp, 2012). Of particular interest, enhanced LPP amplitudes have been observed for neutral expressions of primed familiar faces (Schweinberger et al., 2002; Kaufmann et al., 2008), and when faces are high in social relevance (e.g., romantic partner, family members; Guerra et al., 2012).

The effects of emotional stimulus content on attention have also been documented with a variety of other visual stimuli (e.g., pictures of naturalistic scenes, words, and hand gestures; Schupp et al., 2006a; Kissler et al., 2007; Flaisch et al., 2009; Schacht and Sommer, 2009). Further, EPN and LPP components were found to vary as a function of emotional arousal (i.e., pronounced EPN/LPP for highly emotional arousing pictures; Schupp et al., 2006a), and the LPP appeared sensitive to emotion regulation (Hajcak et al., 2010; Thiruchselvam et al., 2011). In addition, both ERP components have been observed to occur spontaneously while passive picture viewing and during performance of concurrent explicit attention tasks (Schupp et al., 2006a; Pourtois et al., 2013). These results are in line with those of several neuroimaging studies (i.e., showing increased BOLD responses in distributed occipital, parietal and inferior temporal networks; Junghöfer et al., 2006; Sabatinelli et al., 2011) and studies that have shown clear differences in autonomic and reflex activity for emotional compared to neutral stimuli (e.g., Bradley et al., 2001). In sum, there is ample evidence supporting the notion that EPN and LPP components reflect motivationally guided selective attention to significant stimuli (Schupp et al., 2006a).

Building on these findings, the present study examined the joint effects of social relevance of facial stimuli and the displayed emotional expressions. Using an instructional learning paradigm, participants were informed that they would later be introduced to the person presented in a specific face picture. Thus, these faces acquired social relevance by virtue of being potential interaction partners and were contrasted with other non-relevant face actors. Furthermore, the manipulation of facial expressions (happy, neutral, angry) allowed to model the emotional valence and arousal of the anticipated social situation. In light of previous research on face processing, electrocortical processing was hypothesized to differentiate social relevant from non-relevant faces (enhanced EPN/LPP). Further, valence effects are proposed to account for prioritized emotion processing (e.g., threat- or happy-advantage; Schupp et al., 2004; Williams et al., 2006). For instance, based on higher motivational impact, emotional compared to neutral face processing may benefit from additional social relevance as reflected by enhanced LPP amplitudes (Schupp et al., 2007). Integrating different experimental paradigms and methodologies, the present study constitutes a new experimental approach to examine the mutual impact of social and emotional processes by means of the anticipation of a socially relevant situation.

## Methods

### Participants

The sample consisted of 26 healthy volunteers (16 females) aged between 19 and 34 years (*M* = 23, *SD* = 4.3) and recruited from the University of Mannheim (STAI-State *M* = 35.3, *SD* = 4.6; STAI-Trait *M* = 38.8, *SD* = 7.7; SIAS *M* = 16, *SD* = 7.4; FNE-brief version *M* = 33.9, *SD* = 7.8). All participants were informed about the study protocol before providing informed consent in accordance with the university’s ethics guidelines. Participants received course credits for their participation.

### Materials and presentation

**Figure 1:**
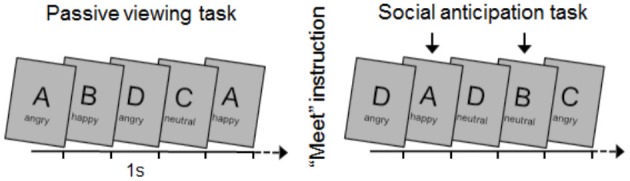
**Illustration of the experimental procedure**. Pictures of 4 face actors (A, B, C, D) displaying happy, neutral, and angry facial expressions were presented randomly (1 s each) in 2 experimental tasks (each 720 trials). Participants were instructed to attend all stimuli in the passive viewing task. Following picture ratings, the main instruction about social contingencies was given: “You are going to meet one of these two people at the end of the experiment” (here indicated by an arrow). Finally, participants again rated face stimuli and were debriefed.

Happy, neutral, and angry facial expressions of 4 different face actors (2 female) were selected from the Karolinska Directed Emotional Faces (KDEF; Lundqvist et al., 1998).^1^ Pictures (1024 × 768 pixels) were randomly presented for 1 s without interstimulus gaps (see Figure 1). The full set of pictures (*N* = 12) was presented 60 times during two separate blocks, each consisting of 720 trials. The first block served as a control condition without specific instructions (passive viewing task). For the second block (meet task), two specific face actors (1 female and 1 male) were introduced as future interaction partners. Accordingly, two face actors were instructed as relevant whereas the other two face actors were non-relevant with respect to future interaction. Assignment of face stimuli to the relevant/non-relevant condition was counterbalanced across participants. Within blocks, face pictures were presented in a different order for each participant. Accounting for potential repetition effects (see Flaisch et al., 2008), picture randomization was restricted to no more than three repetitions of the same facial expression, equal transition probabilities between facial expressions and face actors, and no immediate repetition of the same actor displaying the same facial expression. Pictures were presented on a 22 inch computer screen located approximately 1 meter in front of the participants.

### Procedure

After the EEG sensor net was attached, participants were seated in a dimly-lit and sound-attenuated room. During a practice run (12 picture trials), participants were familiarized with the picture viewing procedure. In the following passive viewing task participants were instructed to attend to each picture appearing on the screen. Before the meet task, instructions were given concerning the relevance of face stimuli by indicating who were the relevant and non-relevant face actors. With respect to the kind of interaction situation, the meet instruction was deliberately kept vague and neutral (“You are going to meet one of these two people at the end of the experiment”). After each block, valence and arousal ratings of the picture set was assessed using the paper-pencil version of the self-assessment manikin (SAM; Bradley and Lang, 1994). At the end of the experiment, a debriefing interview was completed.

### EEG recording

Electrophysiological data were collected using a 64 actiCap system (BrainProducts, Munich, Germany) with Ag/AgCl active electrodes mounted into a cap according to the 10–10 system (Falk Minow Services, Herrsching, Germany). The EEG was recorded continuously with a sampling rate of 500 Hz with FCz as the reference electrode, and filtered on-line from 0.1–100 Hz using VisionRecorder acquisition software and BrainAmp DC amplifiers (BrainProducts, Munich, Germany). Impedances were kept below 10 kΩ. Off-line analyses were performed using VisionAnalyzer 2.0 (BrainProducts) and EMEGS (Peyk et al., 2011) and included low-pass filtering at 30 Hz, artifact detection, sensor interpolation, baseline-correction, and conversion to an average reference (Junghöfer et al., 2000). Stimulus-synchronized epochs were extracted and lasted from 100 ms before to 800 ms after stimulus onset. Finally, separate average waveforms were calculated for the experimental conditions Facial Expression (happy, neutral, angry), Task (passive, meet), and Relevance (relevant, non-relevant),^2^ for each sensor and participant.

### Data reduction and analyses

#### Self-report data

Valence and arousal ratings were analyzed with repeated measures ANOVAs including the factors Facial Expression (happy, neutral, angry), Task (passive, meet), and Relevance (relevant, non-relevant).

#### Event-related potentials

To examine the effects of facial expression, instructed task, and relevance on face processing, a two-step procedure was used. As a first step, visual inspection and single sensor waveform analysis were used in concert to identify relevant ERP components. To this end, single sensor waveform analyses were calculated for each time point and each sensor separately (see Peyk et al., 2011) for the factors Facial Expression (happy, neutral, angry), Task (passive, meet), and Relevance (relevant, non-relevant). To correct for multiple testing, effects were only considered meaningful when the effects were observed for at least eight continuous data points and two neighboring sensors (cf., Bublatzky and Schupp, 2012). Supporting this cluster selection procedure, visual inspection helped ensure that no effects relevant to the main hypothesis regarding the interaction between Facial Expression, Task, and Relevance were missed.

Following this, conventional ERP analyses were based on area scores. Repeated measures ANOVAs based on mean activity in selected sensor clusters and time windows were performed. The P1 component was scored over parieto-occipital cluster (left: O1, PO3; right: O2, PO4) within 100 and 140 ms after picture onset. The N170 was scored at P7 and P8 between 150 and 200 ms. The EPN component was scored at bilateral posterior sensors (PO9 and PO10) between 260 and 360 ms after stimulus onset. To account for the broad distribution of the LPP component, mean activity was scored in bilateral centro-parietal clusters (left: FC1, C1, CP1, P1; right: FC2, C2, CP2, P2) in a time window from 450–700 ms.

An overall multivariate ANOVA tested interaction effects between Facial Expression (happy, neutral, angry), Task (passive, meet), Relevance (relevant, non-relevant), and Laterality (left, right) as a function of ERP Component (P1, N170, EPN, LPP) using Wilks statistics. Significant main effects were observed for Component, *F*_(3,23)_ = 30.72, *p* < 0.001, $\eta_{p}^{2}$ = 0.80, Facial Expression, *F*_(2,24)_ = 11.78, *p* < 0.001, $\eta_{p}^{2}$ = 0.50, Task, *F*_(1,25)_ = 8.51, *p* < 0.01, $\eta_{p}^{2}$ = 0.25, but not for Relevance, *F*_(1,25)_ = 0.15, *p* = 0.70, $\eta_{p}^{2}$ = 0.01, or Laterality, *F*_(1,25)_ = 3.35, *p* = 0.08, $\eta_{p}^{2}$ = 0.12. Of particular importance, higher-order interactions were revealed for Component by Facial Expression, *F*_(6,20)_ = 9.81, *p* < 0.001, $\eta_{p}^{2}$ = 0.75, and Component by Task, *F*_(3,23)_ = 16.69, *p* < 0.001, $\eta_{p}^{2}$ = 0.69. Directly testing the interaction between the three task-sensitive ERP components (P1, EPN, LPP) revealed significant variation of Component as a function of Facial Expression, *F*_(4,22)_ = 13.23, *p* < 0.001, $\eta_{p}^{2}$ = 0.71, and Task, *F*_(2,24)_ = 26.13, *p* < 0.001, $\eta_{p}^{2}$ = 0.69. To follow up on these interactions, separate repeated measures ANOVAs including the factors Facial Expression, Task, Relevance, and Laterality were conducted for each ERP component.

For effects involving repeated measures, the Greenhouse-Geisser procedure was used to correct for violations of sphericity, and as a measure of effect size the partial *η*^2^ ($\eta_{p}^{2}$) are reported. To control for type 1 error, Bonferroni correction was applied for *post hoc* *t*-tests.

## Results

### Self-report data

Overall, valence ratings differed significantly for Facial Expression, *F*_(2,48)_ = 308.06, *p* < 0.001, $\eta_{p}^{2}$ = 0.93. Happy facial expressions (*M* = 7.86, *SD* = 0.16) were rated more pleasant than neutral and angry faces (*M* = 5.15 and 2.44, *SD* = 0.16 and 0.14), *p*s < 0.001, and neutral as more pleasant than angry faces, *p* < 0.001. Although a marginal significant main effect of Task,* F*_(1,24)_ = 3.78, *p* = 0.06, $\eta_{p}^{2}$ = 0.14, indicated that faces were rated as more pleasant during meet task, the interaction Facial Expression by Task was not significant, *F*_(2,48)_ = 1.34,* p* = 0.27, $\eta_{p}^{2}$ = 0.05. Neither instructed Relevance, *F*_(1,24)_ = 0.57, *p* = 0.27, $\eta_{p}^{2}$ = 0.05, nor any higher-order interaction reached significance, *F*s < 1, *p*s > 0.70, $\eta_{p}^{2}$ < 0.01.

Arousal ratings varied for Facial Expression, *F*_(2,48)_ = 45.82, *p* < 0.001, $\eta_{p}^{2}$ = 0.66. Both happy and angry facial expressions (*M* = 4.21 and 5.81, *SD* = 0.38 and 0.33) were rated as more arousing than neutral (*M* = 2.62, *SD* = 0.29), *p*s < 0.001, and angry faces as more arousing than happy expressions, *p* < 0.01. No main effects were observed for Task or Relevance, *F*s_(1,24)_ = 2.79 and 1.67, *p*s = 0.11 and 0.21, $\eta_{p}^{2}$ = 0.10 and 0.07. However, arousal ratings varied as a function of Facial Expression by Task, *F*_(2,48)_ = 6.28, *p* < 0.01, $\eta_{p}^{2}$ = 0.21. To follow up on the differential impact of passive viewing and meet task, facial expressions were tested separately.

Happy face pictures, were rated as more arousing during passive viewing than meet task, Task *F*_(1,24)_ = 14.32, *p* < 0.01, $\eta_{p}^{2}$ = 0.37. Neither Relevance, *F*_(1,24)_ = 0.01, *p* = 0.93, $\eta_{p}^{2}$ < 0.01, nor the interaction Task by Relevance reached significance, *F*_(1,24)_ = 1.08, *p* = 0.31, $\eta_{p}^{2}$ = 0.04. Similarly, angry faces were rated higher in arousal during passive viewing compared to meet task, *F*_(1,24)_ = 7.48, *p* < 0.05, $\eta_{p}^{2}$ = 0.24. Neither Relevance, *F*_(1,24)_ = 3.09, *p* = 0.09, $\eta_{p}^{2}$ = 0.11, nor Task by Relevance, *F*_(1,24)_ = 0.13, *p* = 0.72, $\eta_{p}^{2}$ < 0.01, reached significance. In contrast, arousal ratings for neutral faces did not vary by Task, *F*_(1,24)_ = 1.0, *p* = 0.33, $\eta_{p}^{2}$ = 0.04, Relevance, *F*_(1,24)_ = 0.12, *p* = 0.73, $\eta_{p}^{2}$ = 0.01, or Task by Relevance, *F*_(1,24)_ < 0.01, *p* = 1.0, $\eta_{p}^{2}$ < 0.01.

### Event-related potentials

**Figure 2:**
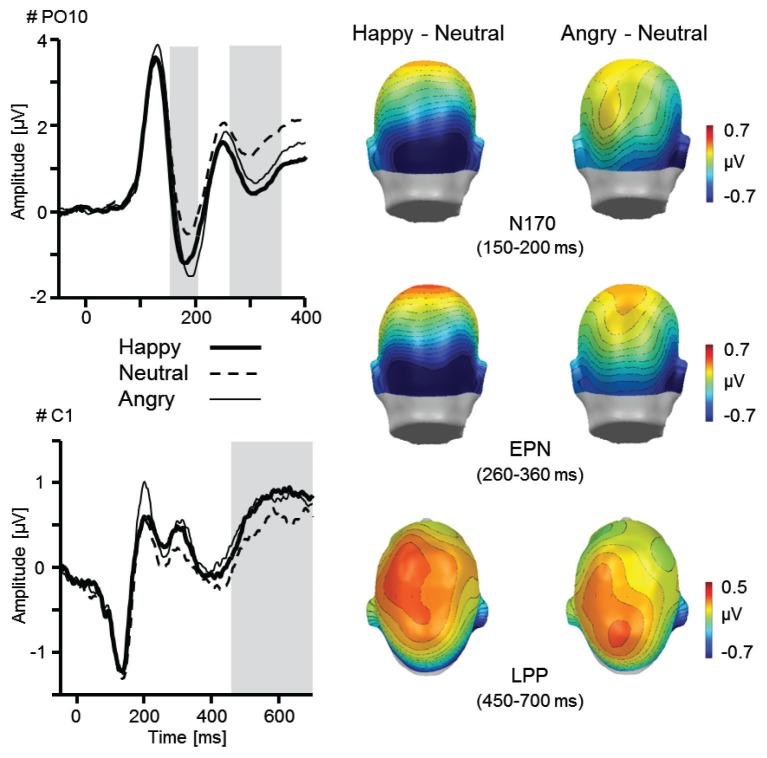
**Illustration of the main effect Facial Expression as revealed by the N170, EPN, and LPP component**. ERP waveforms for an exemplary occipital (PO10) and central sensor (C1) for happy, neutral, and angry faces. Topographical difference maps (happy–neutral, angry–neutral) display the averaged time interval plotted on a back (N170: 150–200 ms; EPN: 260–360 ms) and top view (LPP: 450–700 ms) of a model head. Analyzed time windows are highlighted in gray (PO10: N170 and EPN; C1: LPP).

**Figure 3:**
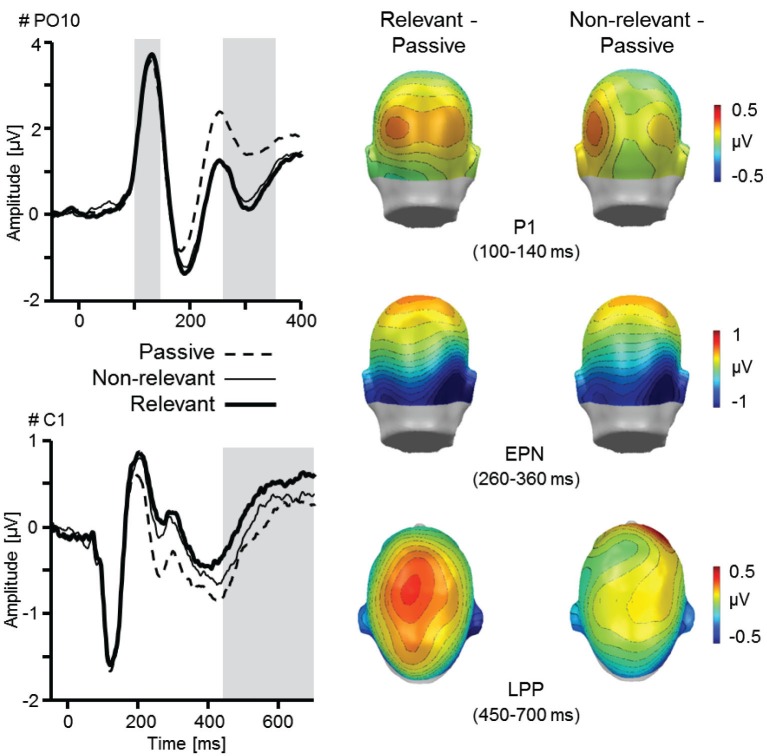
**Illustration of the main effects Task and Relevance as revealed by the P1, EPN, and LPP component**. ERP waveforms for an exemplary occipital (PO10) and central sensor (C1) for relevant and non-relevant face stimuli, each compared to the passive viewing condition. Topographical difference maps (relevant–passive, non-relevant–passive) display the averaged time interval plotted on the back of a model head (P1: 100–140 ms; EPN: 260–360 ms) and a top view (LPP: 450–700 ms). Analyzed time windows are highlighted in gray (PO10: P1 and EPN; C1: LPP).

**Figure 4:**
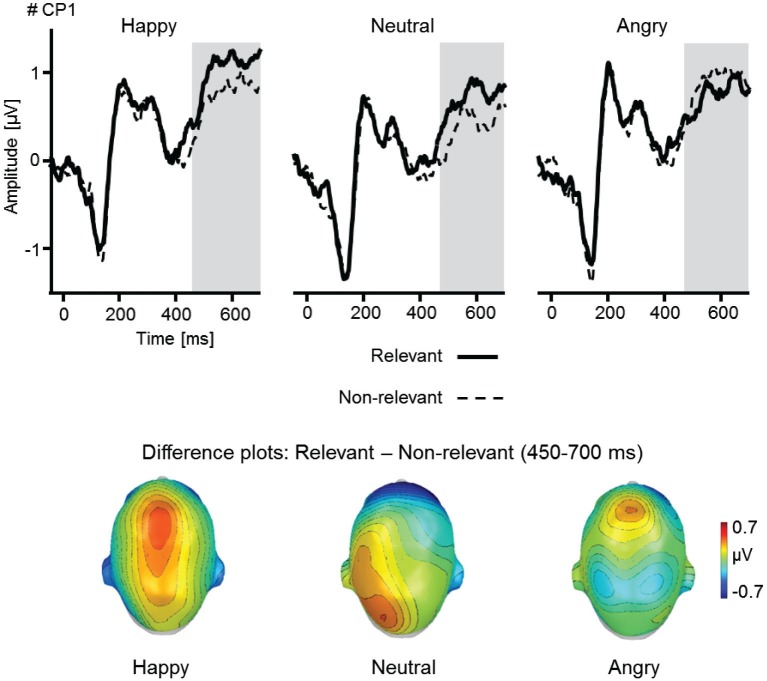
**Illustration of Facial Expression as a function of Relevance as revealed by the LPP component**. ERP waveforms for an exemplary centro-parietal sensor (CP1) for happy, neutral, and angry faces when relevant or non-relevant. Topographical difference maps (relevant–non-relevant) display the averaged time interval (450–700 ms) plotted on a top view of a model head. Analyzed time window for the LPP are highlighted in gray.

Results indicated that verbal instructions about future interaction partners modulated early and late face processing as revealed by enhanced P1, EPN, and LPP amplitudes (Figures 2, 3). Further, the interaction of social and emotional relevance varied across the visual processing stream. Whereas early components revealed independent main effects of Facial Expression and Task (shown by P1, N170, and EPN), the LPP was markedly augmented for happy faces considered as future interaction partners (Figure 4).

#### P1 component

Enhanced P1 amplitude for the meet compared to passive viewing task reached marginal significance, *F*_(1,25)_ = 3.57, *p* = 0.07, $\eta_{p}^{2}$ = 0.13, however, instructed Relevance did not increase P1 amplitude, *F*_(1,25)_ = 0.01, *p* = 0.92, $\eta_{p}^{2}$ < 0.01. Further, emotional Facial Expression modulated the P1 component, *F*_(2,50)_ = 7.44, *p* < 0.01, $\eta_{p}^{2}$ = 0.23. Follow-up tests revealed that amplitudes were more pronounced for angry facial expressions compared to neutral and happy faces, *F*s_(1,25)_ = 8.71 and 10.72, *p*s < 0.01, $\eta_{p}^{2}$ = 0.26 and 0.30. The difference between happy and neutral facial expressions was not statistically significant, *F*_(1,25)_ = 0.70, *p* = 0.41, $\eta_{p}^{2}$ = 0.03. The P1 amplitude was more pronounced over the right hemisphere, *F*_(1,25)_ = 8.79, *p* < 0.01, $\eta_{p}^{2}$ = 0.26. No further interactions including Facial Expression, Task or Relevance reached statistical significance, *F*s < 1.76, *p*s > 0.18, $\eta_{p}^{2}$ < 0.07.

#### N170 component

Whereas Task and Relevance did not modulate the N170, *F*s_(1,25)_ = 0.31 and 0.33, *p* = 0.58 and 0.57, $\eta_{p}^{2}$ = 0.01 and 0.01, amplitudes varied as a function of Facial Expression, *F*_(2,50)_ = 9.23, *p* = 0.001, $\eta_{p}^{2}$ = 0.27. The N170 was more pronounced for both happy and angry faces compared to neutral facial expressions, *F*s_(1,25)_ = 26.70 and 3.98, *p*s < 0.001 and = 0.06, $\eta_{p}^{2}$ = 0.52 and 0.14. The difference between happy and angry faces reached marginal significance, *F*_(1,25)_ = 4.02, *p* = 0.06, $\eta_{p}^{2}$ = 0.14. No main effect of Laterality was observed, *F*_(1,25)_ = 2.75, *p* = 0.11, $\eta_{p}^{2}$ = 0.10, nor any interaction including Facial Expression, Task, and Relevance reached statistical significance, *F*s < 0.71, *p*s > 0.48, $\eta_{p}^{2}$ < 0.03.

#### Early posterior negativity

More pronounced negativity was observed for the meet compared to the passive viewing task, *F*_(1,25)_ = 43.61, *p* < 0.001, $\eta_{p}^{2}$ = 0.64, however, relevance instruction did not modulate the EPN, *F*_(1,25)_ = 0.80, *p* = 0.38, $\eta_{p}^{2}$ = 0.03. Replicating previous findings, the EPN amplitude varied as a function of Facial Expression, *F*_(2,50)_ = 16.28, *p* < 0.001, $\eta_{p}^{2}$ = 0.39. Happy and angry face processing was associated with enlarged EPN amplitudes compared to neutral stimuli, *F*s_(1,25)_ = 37.91 and 10.94, *p* < 0.001 and 0.01, $\eta_{p}^{2}$ = 0.60 and 0.30. Further, the EPN was more pronounced for happy compared to angry faces, *F*_(1,25)_ = 5.05, *p* < 0.05, $\eta_{p}^{2}$ = 0.17. In addition, more pronounced negativities were observed over the left in contrast to the right hemisphere, *F*_(1,25)_ = 12.30, *p* < 0.01, $\eta_{p}^{2}$ = 0.33. No further interactions including Facial Expression, Task, and Relevance reached statistical significance, *F*s < 1.1, *p*s > 0.33, $\eta_{p}^{2}$ < 0.05.

#### Late positive potential

Broadly distributed LPP were modulated by Task, *F*_(1,25)_ = 6.41, *p* < 0.05, $\eta_{p}^{2}$ = 0.20, and Facial Expression, *F*_(2,50)_ = 5.34, *p* = 0.01, $\eta_{p}^{2}$ = 0.18. Happy and angry faces elicited larger LPPs compared to neutral materials, *F*s_(1,25)_ = 11.46 and 5.21, *p*s < 0.01 and 0.05, $\eta_{p}^{2}$ = 0.31 and 0.17, although no difference was found between happy and angry facial expressions, *F*_(1,25)_ = 0.23, *p =* 0.64, $\eta_{p}^{2}$ = 0.01. No differences were observed for instructed Relevance, *F*_(1,25)_ = 1.74, *p =* 0.20, $\eta_{p}^{2}$ = 0.07, and Laterality, *F*_(1,25)_ < 0.01, *p* = 0.98, $\eta_{p}^{2}$ < 0.01.

Of particular interest, a significant interaction emerged for Facial Expression by Relevance, *F*_(2,50)_ = 3.6, *p* < 0.05, $\eta_{p}^{2}$ = 0.12. Further, a near-significant interaction was observed for Task by Relevance, *F*_(2,50)_ = 3.32, *p =* 0.08, $\eta_{p}^{2}$ = 0.12, but not for Facial Expression by Task, *F*_(2,50)_ = 2.18, *p* = 0.14, $\eta_{p}^{2}$ = 0.08, or the higher order interaction Facial Expression by Task by Relevance, *F*_(2,50)_ = 0.08, *p* = 0.91, $\eta_{p}^{2}$ < 0.01. To follow up these interactions, analyses were conducted separately for each experimental task (see Figure 4).

For the meet task, a significant main effect of Facial Expression was observed, *F*_(2,50)_ = 4.86, *p* < 0.05, $\eta_{p}^{2}$ = 0.16. Follow-up analyses revealed pronounced LPP amplitudes for happy faces, *F*_(1,25)_ = 9.33, *p* < 0.01, $\eta_{p}^{2}$ = 0.27, and marginally significant for angry compared to neutral facial expressions, *F*_(1,25)_ = 3.89, *p* = 0.06, $\eta_{p}^{2}$ = 0.14. No difference was observed between happy and angry facial expressions, *F*_(1,25)_ = 1.26, *p =* 0.27, $\eta_{p}^{2}$ = 0.05. Whereas, the interaction Facial Expression by Relevance did not reach significance, *F*_(2,50)_ = 1.83, *p* = 0.18, $\eta_{p}^{2}$ = 0.07, a near-significant main effect of Relevance was observed, *F*_(1,25)_ = 3.85, *p =* 0.06, $\eta_{p}^{2}$ = 0.13. Exploratory follow-up analyses testing relevant compared to non-relevant faces revealed enhanced LPP amplitudes for relevant happy faces, *F*_(1,25)_ = 4.12, *p =* 0.05, $\eta_{p}^{2}$ = 0.14, but not for relevant neutral, *F*_(1,25)_ = 2.32, *p* = 0.14, $\eta_{p}^{2}$ = 0.09, or angry faces, *F*_(1,25)_ = 0.02, *p* = 0.91, $\eta_{p}^{2}$ < 0.01.

In contrast, for the passive viewing task, only the main effect of Facial Expression reached marginal significance, *F*_(2,50)_ = 2.75, *p* = 0.07, $\eta_{p}^{2}$ = 0.10. Follow-up tests revealed pronounced LPP for angry faces, *F*_(1,25)_ = 4.47, *p* < 0.05, $\eta_{p}^{2}$ = 0.15, and marginally enhanced amplitudes for happy faces, *F*_(1,25)_ = 2.92, *p* = 0.10, $\eta_{p}^{2}$ = 0.10, compared to neutral facial expressions. No difference was observed for happy and angry faces in the passive viewing task, *F*_(1,25)_ = 0.35, *p* = 0.56, $\eta_{p}^{2}$ = 0.01. Neither the main effect Relevance, *F*_(1,25)_ < 0.01, *p* = 0.99, $\eta_{p}^{2}$ < 0.01, and Laterality modulated LPP amplitudes, *F*_(1,25)_ = 0.03, *p* = 0.86, $\eta_{p}^{2}$ < 0.01, nor any interaction reached significance, *F*s < 1.86, *p*s > 0.17, $\eta_{p}^{2}$ < 0.07.

## Discussion

The present study examined the impact of instructed social relevance and emotional facial expression on face processing. The main finding was that the mere verbal instruction about social contingencies can modulate early and late face processing as indicated by enhanced P1, EPN, and LPP amplitudes. Importantly, event-related potential measures revealed that the interaction of social and emotional significance varied across visual processing stream. Whereas rather early components revealed independent main effects of facial expression and task instruction (P1, N170, and EPN), the LPP was augmented specifically for happy faces of future interaction partners. These results support the notion of joint impact of emotional and social information mediating face perception.

The anticipation of social interaction with another individual is of considerable value. In the present study, social relevance was manipulated by introducing two specific face actors as future interaction partners (meet task). Results indicate that this socio-emotional context is associated with specific processing patterns as participants view face pictures. The first ERP component sensitive to both task instruction and emotional facial expression was the P1 component, which was enlarged for angry faces compared to happy and neutral facial expressions. Further, regardless of facial expression, enhanced P1 amplitudes were observed during meet compared to passive viewing task. Thus, several previous findings were replicated: enhanced P1 amplitudes in explicit attention tasks (Hillyard and Anllo-Vento, 1998; Pourtois et al., 2004) and implicit processing biases during self-relevant conditions (e.g., instructed threat or in specific phobia; Kolassa et al., 2006; Michalowski et al., 2009; Bublatzky and Schupp, 2012). Presumably based on intensified visual processing in the extrastriate cortex (Hillyard and Anllo-Vento, 1998; Pourtois et al., 2013), the present P1 effects may indicate enhanced vigilance during task conditions of high self-relevance.

Both N170 and EPN components varied as a function of emotional facial expression. As enhanced negativities have been found for both happy and angry compared to neutral faces, these findings suggest that selective face processing occurs as a function of stimulus arousal. Whereas the N170 has been mostly related to structural encoding of non-affective facial features (Sato et al., 2001; Eimer and Holmes, 2002) within occipito-temporal areas (e.g., STS; Itier and Taylor, 2004), the present data are in line with a growing body of literature showing that the N170 is subject to emotional modulation (Pizzagalli et al., 2002; Batty and Taylor, 2003; Rossignol et al., 2005) similar to the EPN component. Further, indicating the enhanced relevance of facial stimuli for (sub-) clinical populations with high levels of social anxiety, pronounced N170 and EPN amplitudes have been observed for angry facial expression (Kolassa and Miltner, 2006; Mühlberger et al., 2009; Wieser et al., 2010). Here, valence specific effects were observed in healthy participants, however, with more pronounced N170/EPN for happy facial expressions. One promising direction for future studies is to manipulate the implicit level of social relevance when examining interindividual differences in emotional face processing (e.g., familiar loved vs. unfamiliar faces displaying emotions; Guerra et al., 2012).

Regarding late positive potentials, face processing was modulated by both task- and emotional relevance. Similar to past research (Schupp et al., 2004), faces displaying angry expressions were associated with enhanced LPP amplitudes, however, this effect was similarly present for happy faces. Of particular interest, the social relevance manipulation revealed an interactive relationship with emotional facial expression. Whereas both happy and angry faces elicited an enhanced late parietal positivity compared to neutral stimuli, this effect was more pronounced when viewing potential interaction partner displaying happy facial expressions. A similar trend was observed for neutral, but not angry, faces of purported interaction partners compared to non-relevant faces. Thus, whereas emotional and social relevance independently modulated early ERP components—indicating either a threat advantage (P1) or selective emotion processing (EPN)—later processing stage revealed specifically enhanced amplitudes for socially relevant happy faces (LPP). These findings appear in line with the evaluative space model of affective processing (Cacioppo and Berntson, 1994; Cacioppo et al., 1999). Depending on the level of activation, emotional input may provoke different processing and response gradients. For instance, at low activation levels, pleasant stimuli may exert a greater influence than unpleasant stimuli in guiding motivational tendencies (e.g., explorative behavior). Accordingly, in rather low-arousing experimental conditions, happy facial expression may be more efficient in activating the motivational approach system than angry faces fostering avoidance. This hypothesis could be tested with socially relevant faces presented under conditions of low and high arousal (e.g., threat-of-shock paradigm; Grillon and Charney, 2011; Bublatzky et al., 2010, 2013). Importantly, future research is needed to connect findings from the perceptual/attentional domain to the functional level, for instance, by testing approach/avoidance behavior (e.g., decision making; Pittig et al., 2014) to socially relevant happy/angry faces in social phobia (Wangelin et al., 2012).

Over and above the impact of implicit stimulus relevance (i.e., emotional facial expression), explicit instructions about social relevance in the meet task was associated with increased P1, EPN, and LPP amplitudes. These findings may complement recent research utilizing selective attention paradigms (Delorme et al., 2004; Pourtois et al., 2013). For instance, Schupp et al. (2007) observed pronounced EPN and enhanced late parietal positivities for target pictures of different semantic categories. Of particular interest, pictures displaying highly arousing content potentiated attention effects specifically during later processing stages (Schupp et al., 2007). In the present social anticipation task, the emotional facial features were no counting targets and actually rated as little arousing; however, a boost of emotion-focused attention was observable specifically for happy facial expression of purported interaction partner. Here, the reference to neural systems involved in various means of relevance processing based on bottom-up or top-down regulation may be informative (e.g., relevance based on task instruction, emotional, or social information; Schmitz and Johnson, 2007; Pourtois et al., 2013). For instance, paying attention to specific stimulus features modulates BOLD responses in the visual cortex (Kastner and Ungerleider, 2000), and for both emotional scenes and facial expressions a great overlap of neural activity has been demonstrated in the amygdala and medial prefrontal cortex (Sabatinelli et al., 2011); the latter being strongly involved in self-referential processing (Gusnard et al., 2001; Northoff et al., 2006; Olsson and Ochsner, 2008; Schwarz et al., 2013).

Several noteworthy aspects and alternative explanations of the present findings need to be acknowledged and should be addressed in future research. First, the critical test of the interaction between social relevance and facial expression was based on the processing of the same face stimuli that differed only in instructed social relevance. This approach has the advantage of ruling out potential effects due to physical differences, as apparent in comparing “social” vs. “non-social” stimuli, however, required that a fixed order of passive viewing task, followed by social meet instruction, was adopted. Thus, excessive stimulus repetitions may have reduced emotion or task effects. However, similar to previous research (Codispoti et al., 2006; Schupp et al., 2006b), neither EPN nor LPP components revealed a reduction of selective emotion processing in the later task. On the contrary, the present LPP amplitudes were generally enhanced during social anticipation task. Furthermore, cognitive processes—such as working memory load or implicit emotion regulation—may have contributed to the absence of enhanced LPP to socially relevant angry faces. For instance, recent studies observed reduced LPP amplitudes to aversive stimuli under working memory load, suggesting that threat processing is contingent on available cognitive resources (MacNamara et al., 2011; Van Dillen and Derks, 2012). Alternatively, implicit emotion regulation may have reduced LPP amplitudes to aversive stimuli as shown in previous studies (Hajcak et al., 2010; Thiruchselvam et al., 2011). Here, future research may implement resource competition (e.g., by means of concurrent tasks or distractor stimuli) and active emotion regulation strategies. This could help clarify how social relevance affects emotional and cognitive processes in face perception.

The effects of selective attention elicited by either implicit emotional or explicitly instructed task relevance have been assessed in previous studies (Schupp et al., 2007; Pourtois et al., 2013). Extending this line of research, the present study utilized a novel approach to manipulate stimulus relevance by introducing specific face actors as future interaction partner. Social relevance was found to modulate face processing differently across visual processing stream. Whereas early ERP components revealed independent effects of social and emotional relevance (P1, N170, EPN), later processing stages were associated with specifically enhanced LPP for happy facial expressions when displayed by future interaction partner. Thus, social relevance may facilitate evaluative face processing according to socio-emotional settings (i.e., future interaction; Fischer and van Kleef, 2010).

## Conflict of interest statement

The authors declare that the research was conducted in the absence of any commercial or financial relationships that could be construed as a potential conflict of interest.

## Footnotes

^1^KDEF identifiers: actor 1: af20has, af20nes, af20ans; actor 2: af25has, af25nes, af25ans; actor 3: am10has, am10nes, am10ans; actor 4: am25has, am25nes, am25ans.

^2^Note: As the passive viewing task did not contain a relevance manipulation, an artificial data split was undertaken to adjust factor structure (i.e., each 1 male and 1 female face actor were assigned artificially to relevant/non-relevant condition).
